# Hot flashes: a review

**DOI:** 10.3389/fmed.2026.1833603

**Published:** 2026-06-25

**Authors:** Sofia Jamal, Amira Elsabagh, Lubna Wani, Mayada Issa

**Affiliations:** 1 Carilion Clinic, Roanoke, VA, United States; 2 Virginia Polytechnic Institute and State University, Blacksburg, VA, United States

**Keywords:** hormone replacement therapy (HRT), hot flashes – menopause, menopause, targeted therapies and diagnostics, womens health

## Abstract

Hot flashes are the most common vasomotor symptom of menopause, affecting over 75% of women and significantly impairing quality of life. These symptoms are a frequent reason women seek medical care, often from primary care physicians. This review summarizes the clinical presentation, underlying pathophysiology, and current management of hot flashes. Clinically, symptoms include episodic heat sensation, flushing, and sweating, often associated with sleep disturbance. Pathophysiology involves estrogen withdrawal and thermoregulatory dysfunction, including a narrowed thermoneutral zone and increased neurokinin B signaling. Management includes lifestyle modifications and complementary therapies with variable evidence. Hormone replacement therapy remains the most effective treatment for appropriate candidates, while nonhormonal pharmacologic and non-pharmacologic approaches are commonly used when estrogen is contraindicated. Emerging targeted therapies offer additional treatment options. Treatment should be individualized based on symptom severity, patient preferences, and risk profile.

## Introduction

Hot flashes are a common vasomotor symptom characterized by a transient sensation of internal heat accompanied by concomitant flushing, perspiration, and discomfort. They are most associated with menopause, affecting more than 75% of women (1, 2). Contemporary understanding links hot flashes primarily to states of estrogen deficiency, an association that is supported by evidence demonstrating that estrogen therapy significantly reduces the frequency and severity of symptoms (2). Consequently, hot flashes may occur in clinical contexts that lower estrogen levels, including chemotherapy-induced ovarian failure and surgical menopause following bilateral oophorectomy. They are also frequently reported among patients receiving endocrine therapies for breast cancer, such as tamoxifen or aromatase inhibitors.

Hot flashes are also reported in men, particularly in the setting of androgen deprivation. Approximately 80% of men receiving gonadotropin-releasing hormone (GnRH) agonists experience hot flashes, with nearly one quarter identifying them as the most distressing adverse effect of therapy (3). However, hot flashes have also been observed in the general male population. In a Swedish population-based study of non-castrated men aged 55 years and older, 33% reported experiencing hot flashes, and approximately half of those affected described them as bothersome (4).

Although not life-threatening, hot flashes can substantially impair quality of life and daily functioning. Given the broad population affected, recognition and effective management of these symptoms remain important considerations in outpatient care.

### Clinical presentation and risk factors

Patients experiencing hot flashes report a sudden sensation of heat involving the upper torso, often accompanied by flushing and sweating. Individual episodes typically last from several minutes to, in some cases, up to an hour. Symptoms may occur with variable frequency and can arise at any time during the day or night. The sensation of heat is associated with increases in skin temperature and cutaneous blood flow, reflecting underlying peripheral vasodilation in symptomatic regions (2, 5). Nocturnal hot flashes may significantly affect sleep quality. Individuals experiencing nighttime vasomotor symptoms have been shown to experience more frequent awakenings and sleep disruption compared with asymptomatic individuals (6).

Beyond effects on quality of life, evidence suggests that vasomotor symptoms may be associated with adverse long-term health outcomes. Data from the Study of Women’s Health Across the Nation (SWAN) demonstrated that frequent and persistent vasomotor symptoms were associated with an increased risk of subsequent cardiovascular disease events (7). Additional studies have linked hot flashes, particularly nocturnal symptoms, with markers associated with cerebrovascular disease and Alzheimer disease risk (8, 9). Although causality remains unclear, these findings suggest that hot flashes may not be entirely benign symptoms and may identify patients who warrant broader cardiovascular and cognitive risk assessment.

Several factors have been associated with an increased frequency of hot flashes. These include cigarette smoking, lower body mass index (BMI) in men, higher BMI in women, and exposure to warm environmental conditions. Additionally, premenstrual syndrome (PMS) has been identified as a potential risk factor. In one study, 83.4% of reproductive-age women with regular menstrual cycles who experienced PMS reported menopausal-like hot flashes (5). Similarly, Guthrie et al. found that women with PMS were 1.9 times more likely to develop hot flashes within 3 years prior to menopause compared with women without PMS (10).

The prevalence of hot flashes also varies across racial and ethnic groups. Findings from the SWAN study demonstrated the highest incidence among African American women (11). Overall, vasomotor symptoms are associated with wellbeing, fatigue, sleep disturbance, and reduced productivity.

### Role of estrogen and thermoregulation in hot flashes

Although these symptoms in women start around the time of decrease in estrogen and increase in LH pulses, studies have shown that there is no correlation between the levels of these hormones and the occurrence of hot flashes (4, 12). The precise role of estrogen in the pathophysiology of hot flashes remains incompletely understood. Evidence suggests that estrogen withdrawal, rather than absolute estrogen deficiency, may play a key role in triggering hot flashes. This hypothesis is supported by observations that symptoms are often most frequent during the early menopausal transition, when hormonal fluctuations are greatest, and tend to decline later as physiologic adaptation occurs (1, 13). Additional evidence comes from clinical observations that hot flashes do not typically occur in individuals with lifelong absence of estrogen but may develop when exogenous estrogen therapy is initiated and subsequently discontinued (13). Nevertheless, estrogen deficiency alone does not fully explain the phenomenon, as circulating estrogen levels do not consistently differ between symptomatic and asymptomatic individuals (14).

Alterations in thermoregulation may play an important role in the etiology of hot flashes. Studies have demonstrated that increased sympathetic nervous system activity, reflected by elevated norepinephrine levels, can raise core body temperature and precipitate vasomotor symptoms. It has been proposed that individuals who experience hot flashes have a narrowed thermoneutral zone, defined as the range of core body temperatures within which thermoregulatory responses such as sweating or shivering are not activated (15). Research suggests that small temperature elevations preceding hot flashes acting within a reduced thermoneutral zone constitute the triggering mechanism (14). As the upper threshold is passed, the patient will experience increased blood flow, inducing sweat. This will have a cooling effect on the skin, thus hitting the lower threshold of the thermoregulation zone and inducing shivering.

**Figure 1:**
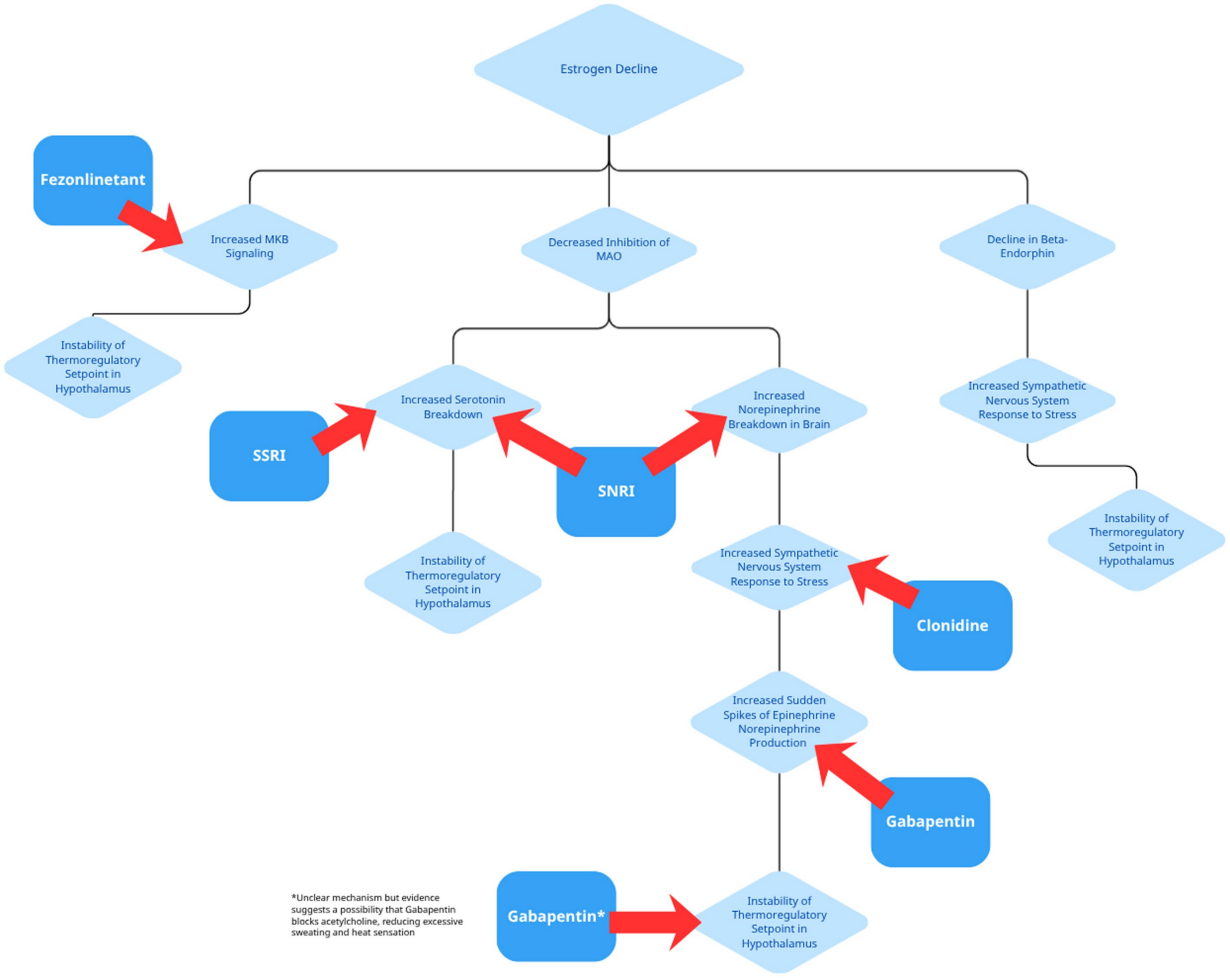
Mechanisms underlying menopausal vasomotor symptoms and targets of nonhormonal treatment. Red arrows indicate the proposed sites of action of nonhormonal therapies.

Recent studies showed that estrogen inhibits neurokinin-3 receptors thus preventing the stimulation of those neurons in the thermoregulatory center in the hypothalamus. As the level of estrogen declines, these receptors will become overstimulated by a neuropeptide named neurokinin B. Hypothalamic NKB expression increases in menopause and has also been noted to be increased after mammalian ovariectomy (16). The proposed mechanisms linking estrogen decline to thermoregulatory instability and downstream neurotransmitter effects are illustrated in Figure 1.

### Complementary and non-prescription therapies

Nonpharmacological management of hot flashes includes lifestyle modifications, behavioral therapies, and mind–body interventions. Cognitive behavioral therapy (CBT) has demonstrated benefit in reducing the perceived burden and interference associated with vasomotor symptoms, with randomized trials showing improvements in hot flash problem ratings, mood, and quality of life (17). Exercise interventions have shown mixed effects on vasomotor symptom frequency, although some studies demonstrated modest improvements in sleep and depressive symptoms (18). A systematic review and meta-analysis including 13 randomized controlled trials found that yoga may improve vasomotor, psychological, somatic, and urogenital menopausal symptoms (19). Acupuncture has also been extensively studied; although, a randomized sham-controlled trial found no significant difference between acupuncture and sham acupuncture in reducing hot flash scores, suggesting that placebo effects may contribute to observed benefit (20). Various herbal supplements, including black cohosh, primrose oil, ashwagandha, *Rheum rhaponticum*, saffron, and phytoestrogen have been studied and used for treatment of hot flashes; however, the lack of regulation for supplements and mixed study results limits the safety and efficacy for recommending use to patients. The market for complementary and alternative therapies is substantial; the estimated cost for a six-month supply of common alternative therapies ranges from $129 to $348 (21).

Some herbal supplements have shown more consistent benefit in the literature. Ashwagandha, *Withania somnifera*, have been shown in two randomized, double-blind, placebo-controlled studies to be effective in relieving hot flash symptoms (22, 23). A systematic review and meta-analysis study on standardized *R. rhaponticum* root extract, Err731, indicates that supplementation is effective in relieving menopausal symptoms thought to be secondary to its specific binding of the ER-*β* receptors (24).

Other supplements have had mixed findings noted through the literature. Two randomized trials studying black cohosh showed no significant effect verses placebo in regards to the number and intensity of hot flashes (25, 26). However, a comparable study of black cohosh and primrose oil showed that both were beneficial in reducing the severity of symptoms with black cohosh also able to reduce frequency (27). Primrose oil has also been shown to have no significant effect on symptoms in a randomized control trial (28). *Crocus sativus*, saffron, has also shown mixed effectiveness in double blind, randomized, placebo-controlled trials. One such trial concludes that saffron is a safe and effective treatment in improving hot flashes while another showed that through saffron had beneficial effects on depression and anxiety symptoms, there was no significant difference in vasomotor or other somatic symptoms in comparison to placebo (29, 30).

### Pharmacologic therapies

**Figure 2:**
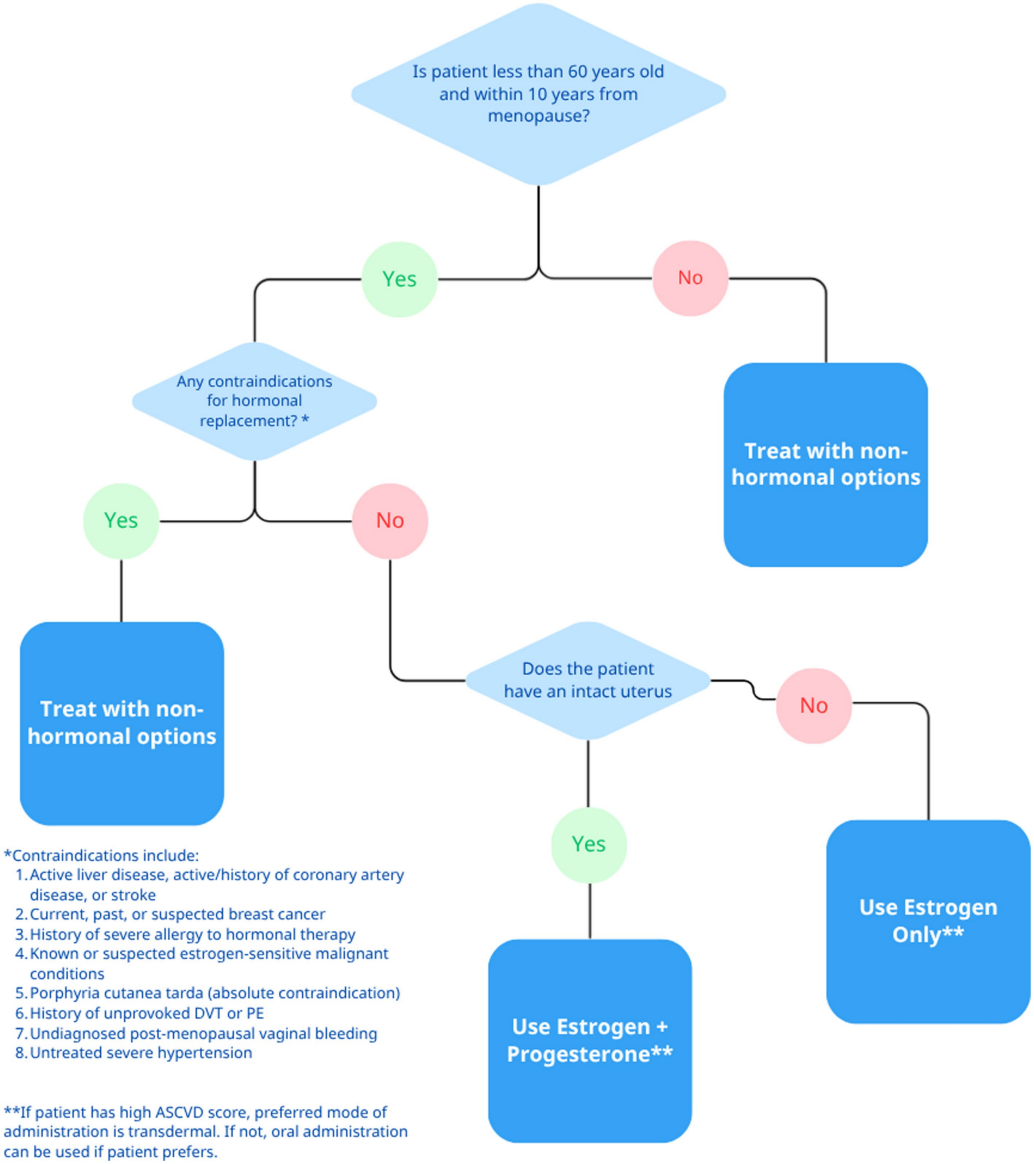
Clinical decision pathway for selection of hormonal and nonhormonal therapies for menopausal vasomotor symptoms.

Women tend to seek pharmacologic and nonprescription options to alleviate these symptoms due to their negative impact on quality of life. Management of vasomotor symptoms is guided by patient age, time since menopause, and the presence of contraindications to hormone therapy. A simplified clinical approach to treatment selection is summarized in Figure 2. Hormone replacement therapy (HRT) was widely used prior to the publication of the Women’s Health Initiative Study (WHI) after which their use declined. Current guidelines identify hormone therapy as the most effective treatment for moderate to severe vasomotor symptoms in appropriately selected patients, particularly in women younger than 60 years or within 10 years of menopause onset (31). Treatment should be individualized based on symptom severity and patient risk factors, using the lowest effective dose with periodic reassessment of risks and benefits. Currently it is indicated to alleviate moderate to severe vasomotor symptoms if used within the first 5 years of menopause. In patients with an intact uterus, combined estrogen–progestin therapy is recommended to prevent endometrial hyperplasia, whereas estrogen alone may be used in individuals who have undergone hysterectomy. Hormone therapy is contraindicated in patients with a history of breast cancer, venous thromboembolism, thrombophilia, coronary artery disease, stroke, chronic liver disease, or migraine with aura. Transdermal formulations may be preferred in patients at increased risk of venous thromboembolism due to lower associated thrombotic risk compared with oral formulations (31). The combination of conjugated estrogen with the selective estrogen receptor modulator Bazedoxifene has demonstrated efficacy in reducing vasomotor symptoms while potentially minimizing adverse effects such as breast tenderness and the risk of endometrial stimulation (32, 33).

Nonhormonal pharmacologic therapies are frequently considered for individuals who cannot use estrogen. Because mood changes and sleep disturbances commonly accompany menopause, selective serotonin reuptake inhibitors (SSRIs) and serotonin–norepinephrine reuptake inhibitors (SNRIs) have been evaluated for symptom control. A review by Stubbs et al. reported that Citalopram, Escitalopram, and Paroxetine were effective in reducing the frequency and severity of hot flashes. However, SSRIs may interact with Tamoxifen by altering its metabolism and are generally avoided in patients receiving this therapy for breast cancer. In such cases, SNRIs such as Venlafaxine and Desvenlafaxine are commonly recommended. Venlafaxine has been reported to provide the greatest reduction in symptoms. Gabapentin is another option for patients receiving tamoxifen, although it appears less effective than venlafaxine in reducing hot flash frequency (32% versus 68%) (34).

The α2-adrenergic agonist Clonidine has also been used for vasomotor symptom management. Reduced estrogen levels may alter α2-adrenergic receptor activity, leading to increased sympathetic activation and thermoregulatory instability, which provides a potential explanation for clonidine’s therapeutic effect. However, its clinical utility is limited by variable efficacy and a relatively high incidence of adverse effects, as reported in a meta-analysis by Nelson et al. (35).

Other agents have also been investigated. Extended-release Oxybutynin, commonly used in the treatment of overactive bladder, demonstrated reductions in both the frequency and severity of moderate to severe vasomotor symptoms compared with placebo in a randomized clinical trial (36).

### Targeted therapies

Neurokinin receptor antagonists targeting hypothalamic thermoregulatory pathways have emerged as novel treatment options. Fezolinetant received approval from the U.S. Food and Drug Administration in 2023 for the treatment of moderate to severe vasomotor symptoms. This selective neurokinin-3 receptor antagonist has demonstrated efficacy in reducing hot flash frequency and severity. However, elevations in liver enzymes have been observed, and monitoring of hepatic function every 3 months during the first year of treatment is recommended (37).

Another agent, Elinzanetant, which antagonizes both neurokinin-1 and neurokinin-3 receptors, received FDA approval in 2025. Clinical studies suggest that this therapy may improve sleep disturbance and mood in addition to reducing vasomotor symptoms. Reported adverse effects include headache and somnolence, while elevations in liver enzymes appear to occur less frequently; liver function testing is recommended approximately 3 months after treatment initiation (38).

It is important to note that nonhormonal therapies do not address other manifestations of estrogen deficiency, such as bone loss or genitourinary symptoms. Therefore, treatment selection should be individualized and based on patient preferences, symptom severity, and underlying risk factors, with shared decision-making between the patient and clinician guiding management.

## Conclusion

Hot flashes represent a prevalent and clinically significant manifestation of hormonal and thermoregulatory dysregulation, with a growing understanding of their underlying neuroendocrine mechanisms. While hormone replacement therapy remains the most effective treatment for appropriate candidates, a range of nonhormonal pharmacologic and nonpharmacologic options provide meaningful alternatives for patients with contraindications or preferences against estrogen use. The emergence of targeted therapies, particularly neurokinin receptor antagonists, highlights important progress in mechanism-based treatment. However, variability in treatment response and limitations in long-term safety data underscore the need for further clinical studies and the development of standardized, evidence-based treatment guidelines to optimize patient-centered care.
